# Research on Flexible Sensors for Wearable Devices: A Review

**DOI:** 10.3390/nano15070520

**Published:** 2025-03-30

**Authors:** Jihong Liu, Hongming Liu

**Affiliations:** 1 College of Information Science and Engineering, Northeastern University, Shenyang 110819, China; 2 College of Materials Science and Engineering, Northeastern University, Shenyang 110819, China; 20234401@stu.neu.edu.cn

**Keywords:** wearable devices, smart devices, flexible sensors, real-time feedback, longtime monitoring, biosignal

## Abstract

With the development of new materials and the trend of miniaturization of smart devices, wearable devices are playing an increasingly important role in people’s lives and occupying a larger market share. Meanwhile, the operation of wearable devices is based on the flexible sensors inside them. Although the development of flexible sensors has been very rapid in the more than 20 years since entering the 21st century, facing the booming market and demand at present, the development of flexible sensors still faces many challenges such as more miniaturization, higher integration, greater sustainability, high precision, and more efficient energy saving. This paper aims to summarize the development of flexible sensors, look forward to the future development of such devices, and provide a reference for researchers.

## 1. Introduction

Today, the intelligence and interactivity of electronic devices have gradually become the mainstream trend. The interactivity between devices and users signifies the level of intelligence of a product. The first step for devices to interact with the outside world and users is to collect external information through sensors (in wearable devices, this is often the case where sensors continuously collect various biological signals from users and provide feedback) [1]. Therefore, sensors are the hardware foundation of the entire wearable device.

This review will introduce the use of sensors in the monitoring of biological signals and wearable devices, especially the advantages and development of flexible sensors in high-precision tasks and rapid feedback tasks. Meanwhile, flexible sensors still face many challenges, such as further miniaturization, improvement of anti-interference capabilities, and further enhancement of energy efficiency. These advancements are closely related to other cutting-edge fields such as electrochemistry, microelectronics, and materials science.

This paper will elaborate on the following several aspects:

Firstly, we will focus on the development of wearable devices in recent years. With the advancement of the integrated circuit industry and cutting-edge science, the miniaturization of smart devices has directly led to the rapid development of wearable devices [2,3,4]. Meanwhile, the recent results in material also led to the development of these devices [5,6]. Overall, although wearable devices have made significant technological progress, they still have substantial room for improvement and face challenges in further miniaturization, sustainability, cost reduction, and energy efficiency [2,7,8]. Therefore, this paper will summarize the achievements and challenges in the field of wearable devices.

Due to the unique nature of wearable devices—which involve integration and the monitoring of various parts of the human body—the sensors embedded within them must necessarily meet the requirements of being deformable, stretchable, and durable [9]. Therefore, we will next focus on the core of wearable device hardware—flexible sensors [7]. As is well known, the types of flexible sensors are diverse, including acoustic sensors, optical sensors, piezoelectric sensors, and other more complex products [8,10,11,12]. Through the combination of various types of sensors and processors, the functionality of a complete wearable device is achieved. Micro-electro-mechanical Systems (MEMS) is a prime example of this [8,12,13].

When it comes to the applications of wearable devices and flexible sensors, people’s first thought is often the biomedical/health field, and this is indeed the case. This paper will focus on the applications of wearable devices and flexible sensors in human health monitoring and bio-signal detection [7,11,14,15,16]. For example, E-skin products can be attached to the surface of the human body [17,18,19], which can monitor the pressure on the skin of users. There are also mechanical sensors that detect human motion and gestures by measuring displacement and acceleration [20,21,22]. Currently, devices for monitoring sleep are also very common. These devices typically contain a variety of sensors that assess sleep conditions by continuously monitoring the user’s heart rate, respiratory rate, blood pressure, temperature, and other indicators in real time [15,23,24,25,26]. In addition, devices that assist users in evaluating their mental stress and psychological health through more complex mechanisms are also under development [15].

The development of all the above devices is based on the advancement of functional materials. In recent years, the development of nanomaterials and new structures has provided some mechanisms for sensing technology [5,23,25,27,28]. The development in materials includes numerous advancements in inorganic materials, metallic materials, and graphite-based materials that have directly propelled the development of sensing technologies [29,30,31,32,33,34,35].

The following sections of this paper will elaborate on the above-mentioned four aspects.

## 2. Development of Wearable Devices

### 2.1. Common WHMS in the Market

Currently, there are many commercially available wearable health-monitoring systems on the market. For example, fingertip pulse oximeters provided by manufacturers, heart rate monitors from Omron, and devices like SenseWear Armband [7]. Now, we can consider the most representative and “prevailing” systems (which are called WHMS) in the market.

### 2.2. The Development of E-Skin Devices

In addition to miniaturized wearable devices, there are also larger wearable devices suitable for daily use, such as the SmartShirt system from Sensatex: This is a T-shirt-based wearable system that uses conductive fiber sensors to measure electrocardiogram (ECG), respiration rate, and blood pressure [7,36]. These devices are skin-conforming and can measure various physiological indicators in real time. This skin-conforming nature is similar to that of E-skin and other skin-attached devices [29,37]. These “wearable electronic skins” can accurately detect physiological indicators [38]. The fundamental technical key lies in the mass-producible elastic transistor arrays [29,37]. These devices can self-repair after damage or can be recycled after use. For example, electronic skin made from dynamic covalent thermosetting nanocomposites is repairable, fully recyclable, and moldable. Such characteristics are based on the development of nanoscience [10,20,39]. Such devices are also envisioned to achieve breakthroughs in low energy consumption, capable of harvesting and storing heat emitted from the skin as a backup energy source for the device [37].

### 2.3. Wearable Devices Based on Acoustic Principles

MEMS (Micro-electro-mechanical Systems) also have many applications in the field of wearable devices [40]. AlN (Aluminum Nitride) MEMS acoustic sensing devices are used to monitor heart sounds and accurately detect voice and sound [41]. These devices are encapsulated with silicon polymer and have the characteristics of being lightweight, sweat-resistant, noise-resistant, and having good stability [12,42]. They have achieved a signal-to-noise ratio (SNR) of 72 dBA and a sensitivity of −38 dBFS. This device significantly outperforms traditional stethoscopes in terms of signal-to-noise ratio (SNR), making it suitable for use as a portable, long-term stethoscope for monitoring heartbeats and breathing. MEMS microphones are not only used in the field of biometric monitoring but also serve as high-performance accessories in other portable devices such as smartphones, smartwatches, fitness bands, and smart glasses [41,43]. Since their initial market acceptance in 2003, MEMS microphones have become the dominant product in hearing health devices. They offer improved electroacoustic performance, reliability, and repeatability at a lower cost compared to electret microphones. Currently, MEMS microphones provide noise levels below 25 dBA, handle sound levels exceeding 135 dB, and come in packages smaller than 3.5 mm in length. In addition to microphones, there are also many high-performance acoustic wearable devices with output capabilities, such as MEMS speakers. These speakers feature a small carbon footprint, lightweight, low power consumption, low-cost batch manufacturing compatibility, and improved compatibility with application-specific integrated circuits (ASICs). Currently, these speakers are widely installed in earphones and hearing aids [41]. However, it is also necessary to recognize the current shortcomings of miniature microphones: the diaphragm in the microphone has residual stress after leaving the factory. The stress of the diaphragm can affect the sensitivity and performance of the microphone (especially at sound pressure levels between 20 μPa and 20 Pa) [44], so this is an urgent problem to be solved.

A low-cost structural innovation can significantly improve the residual stress effect—introducing slits into the diaphragm structure [44]. Another intuitive improvement involves incorporating microstructures similar to springs. A semi-constrained polysilicon diaphragm can be supported on a backplate by central and eight peripheral bumps. This design reduces the impact of residual film stress through the flexible spring structure [45]. However, MEMS-based acoustic wearable devices are not the only type of such devices; microphones based on capacitive or piezoelectric principles also hold significant positions. Capacitive devices are used for listening; piezoelectric microphones are more suitable for detecting noise caused by tiny vibrations [42]. In addition to auditory and tactile wearable devices, vision-related devices that meet human needs have also gained significant recognition in the market and technology sectors. The most well-known example is smart glasses based on MEMS. The main technological focus lies in fabricating millions of metal micromirrors on the surface of glass and arranging these mirrors in an array driven by electrostatic forces to improve the optical performance of traditional lenses (typically manifested as a reduction in optical artifacts) [46].

**Figure 1:**
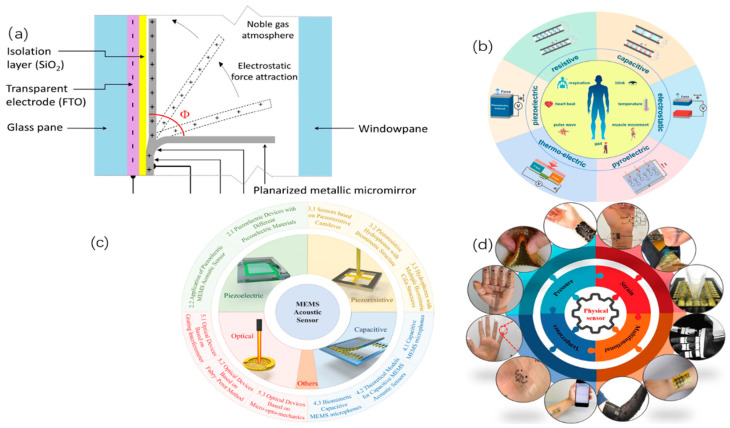
Shows the application of flexible sensors. (**a**) Shows the profile view of a single micromirror [46]. (**b**) Includes the functionalities of wearable multi-functional sensors [4]. (**c**) The classification of acoustic sensors based on different principles [12]. (**d**) Typical applications of flexible sensors [37].

The above, respectively, represent the development and challenges of wearable devices in the areas of vision, touch, and auditory. The details can be seen in Figure 1.

## 3. The Role of Flexible Sensors in Wearable Devices

### 3.1. Subsection

Here, we introduce typical applications of flexible sensors in wearable devices.

**Figure 2:**
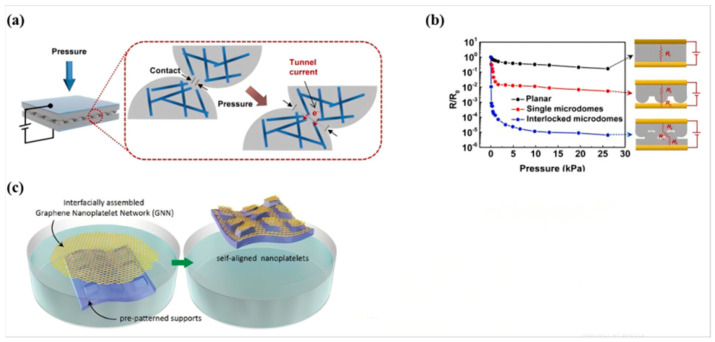
The diagram of mechanism of pressure sensor: (**a**) the diagram of working mechanism; (**b**) these curves show the sensitivity according to the different structures; (**c**) the new transfer technique [17].

In devices based on tactile/skin contact, there are many common sensors, such as pressure sensors that conform to the skin [3,17,30,33,37,47,48,49], acceleration/displacement sensors fixed on human limbs [20,34,50], and temperature (body temperature) sensors [23,24,47,51]. Since these sensors need to conform to the surface of the human body or robotic body, they should possess wear resistance, chemical resistance, mechanical flexibility, and stretchability to adhere to surfaces with three-dimensional curvatures similar to those of human fingertips [17,52]. Currently, the industry is focusing on improving the sensitivity of pressure sensors. The working mechanism can be seen in Figure 2.

Worth mentioning is a commercial submillimeter pressure sensor. This type of sensor is not directly attached to the skin but is instead a vascular pressure sensor attached to the inner walls of blood vessels. Compared to common flexible sensors, the target product requires biocompatibility and more extreme miniaturization [53].

In addition, the research team has developed a prototype electronic circuit for conditioning and reading pressure signals (with a power consumption of less than 14.5 mW in continuous operation mode). To meet biocompatibility requirements, the team also conducted in vivo validation of the product. During the in vivo validation, the sensor successfully recorded the arterial pressure waveform of a female sheep [53]. Although the progress in the laboratory has been successful, further long-term validation is still required after small-scale production.

### 3.2. Temperature Sensors in Wearable Devices

As wearable devices, temperature (room temperature or body temperature) is an important measurement metric. In response to this need, temperature sensors have emerged. Currently, mainstream temperature sensors are primarily based on two types of hardware: photosensitive liquid crystals and resistance.

First, let us introduce temperature sensors based on photosensitive principles and chiral liquid crystals. Traditional infrared temperature sensors have advantages for integration into smart devices, but they require the surface of the object being measured to remain uncovered [23]; otherwise, significant errors may occur. To address this limitation, wearable sensors with temperature visualization capabilities can be used. These devices achieve real-time perception of temperature changes through seamless contact with human skin, enabled by thermochromic materials that change color with temperature. Additionally, digital image processing technology has been used to establish a temperature–color curve within a certain range, with relatively ideal errors: between 26 and 34 degrees Celsius (the most common working environment for such devices), the measurement error is around 0.1 degrees Celsius, while near 40 degrees Celsius, the error increases to approximately 0.4 degrees Celsius [24].

E-tattoo is a temperature sensor based on resistance changes. Its material is a composite of graphene/silk fibroin/calcium ions. Due to changes in electron hopping at the interfaces between adjacent graphene layers, the resistance of E-tattoos varies with temperature. The fundamental reason is the change in resistance caused by the varying rates of water evaporation on the skin surface at different temperatures.

Sensors that detect displacement/acceleration/deformation for human motion/gesture recognition also play a significant role in wearable devices [6,20,54].

### 3.3. Motion Gesture Recognition Sensors in Wearable Devices

First, let us introduce a gesture sensor based on visual recognition that offers rapid response and high sensitivity. This sensor is composed of a multi-walled carbon nanotube (MWCNT) network embedded in a silicone elastomer and is manufactured using laser technology. The laser-fabricated sensor exhibits high tensile sensitivity, a tensile strength of 963 kPa, and a fast strain response time (1 ms). These characteristics, combined with the material’s high electrical conductivity, enable gesture recognition accuracy of up to 94–95%. The principle behind this sensor’s gesture recognition is based on visual recognition, requiring complex mathematical models to construct a three-dimensional hand model, which increases computational complexity [22,55,56,57].

**Figure 3:**
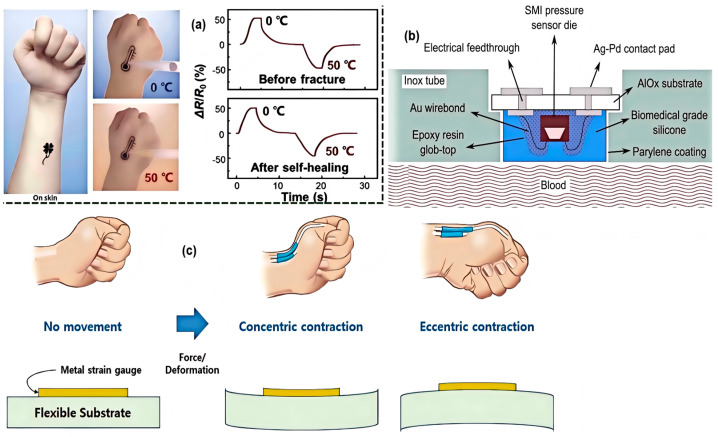
The diagram of mechanisms of some bio-sensors. (**a**) The illustration of the fabrication process [24]. (**b**) The schematic overview of the sensor embedded in the tube [53]. (**c**) Change in strain and resistance caused by the movement of the wrist [56].

Therefore, monitoring devices based on strain and resistance may be a more cost-effective alternative [6,11,58,59,60]. Flexible Epidermal Tactile Sensor Arrays (FETSA) based on strain gauges detect skin/joint deformations, which are converted into electrical signals. By measuring these electrical signals, devices equipped with such sensors can obtain specific motion parameters and, based on these, estimate motion types and trajectories, thereby recognizing user gestures [56]. Specifically, strain gauges are placed on a flexible substrate. Based on wrist movements, the resistance values of the flexible array sensor are used for gesture recognition. The above details can be seen in Figure 3.

### 3.4. Humidity Sensors in Wearable Devices

Owing to the increasing integration of functionalities in wearable devices, other sensors such as humidity sensors are also required [61,62,63].

This study introduces a humidity sensor composed of silver electrodes and an active layer based on carbon nanotubes (CNTs). Specifically, the active layer is fabricated by dispersing CNTs in polyvinyl alcohol (PVA). When humidity increases, the PVA in the active layer absorbs water and swells, causing the spacing between CNTs to increase, which in turn leads to an increase in electrical resistance. Conversely, when humidity decreases, the opposite effect occurs [62].

In general, when discussing wearable devices, it is essential to address flexible sensors. As the fundamental hardware of wearable devices, flexible sensors are capable of receiving mechanical, thermal, and optical signals and converting them into electrical signals, thereby functioning as the central part of the device. By integrating other components such as gyroscopes, communication modules [64], control modules, power sources, and displays into the device, complete functionality can be achieved [7].

## 4. Flexible Sensors in Biomedical Signs Monitoring

The primary function of wearable devices is to monitor biological signals and provide references and assistance for health protection. Flexible sensors play a crucial role in this context. In this section, we focus on the role and progress of flexible sensors in the detection of biological signals and health protection.

### 4.1. Flexible Sensors for Human Motion Detection

We first introduce a device that issues fall alerts to users by detecting human movements [65]. The flexible sensors contained in the device can perceive the activities of human joints and even subtle vibrations [6,66,67]. The specific principle is as follows: Flexible sensors can deform with joint movements and output electrical signals accordingly. For mechanical data such as velocity and acceleration [68], linear motors are required to read the data. To achieve complete early warning functions, software work is also needed, such as designing user interaction systems and communication systems.

During walking, gait detection can also provide users with reminders to prevent falls or accidental injuries [2]. When humans walk, many related pieces of information are periodic, such as frequency, amplitude, peak intervals, and duration [69]. By leveraging these characteristics and capturing valuable information like step count, stride length, walking speed, and gait asymmetry, combined with machine learning tools, it is possible to make highly accurate judgments on gait patterns [70]. This approach can also provide early warnings for conditions such as Parkinson’s disease episodes, knee injuries, and falls [2,39].

Meanwhile, flexible sensors based on mechanical pressure also have applications in the detection of joint movement and limb activity [71,72,73,74]. Flexible sensors composed of a 3D conductive network are highly sensitive strain sensors, with their sensitivity originating from the conductive network structure that allows relative slipping. The sensor exhibits an ideal gauge factor (GF) of 23.18 within a strain range of 25%. The strain caused by joint movement is primarily concentrated on the posterior side, which is transmitted to the sensor and results in changes in its resistance. Significant bending leads to rapid changes in resistance. When the bending angles are 15° and 45°, respectively, the change rate can increase from approximately 1 to 5. Similar phenomena were also observed when examining the movements of the elbow and knee. When the strain is released, the change rate of the sensor’s resistance returns to zero, indicating that the sensor has a rapid response to minor joint movements [73].

### 4.2. Flexible Sensors for Sleep and Breath Monitoring

The psychological stress and sleep issues of modern people have become a focus of health protection. We introduce the sensors in a device that can improve sleep and mental stress [15]. Traditional pressure sensors are unable to achieve both precise response and a wide input range simultaneously. However, the Hybrid Pressure Sensor (HPS) effectively addresses this limitation. The HPS is based on the coupling effect of the triboelectric effect and electrostatic induction and is composed of two types of triboelectric materials with opposite polarities. It features high sensitivity (2.46 V/10^4^ Pa, over the range of 50–600 kPa), a wide response range (up to 1200 kPa), a low detection limit (10 kPa), and high stability over more than 100,000 cycles. Next, we introduce the specific working mechanism of the sensor: By monitoring the pressure changes on the head and shoulders on the pillow and mattress, different sleeping postures can be accurately distinguished. The sensor measures the pressure changes on the shoulders during sleep to reflect the breathing conditions under different sleeping postures [75], thereby effectively distinguishing four different types of sleep breathing behaviors.

In addition to detecting respiration through mechanical methods, breathing can also be monitored by detecting changes in ambient humidity. This is because the humidity of exhaled air is significantly higher than that of the surrounding environment, while inhalation reduces the humidity around the mouth and nose. The impedance of humidity sensors is highly sensitive to changes in humidity. Therefore, incorporating flexible humidity sensors into wearable devices can provide respiration curves [51,76]. By employing the above methods, the device can comprehensively record the user’s sleep information and provide relevant suggestions.

### 4.3. Flexible Sensors for Monitoring User’s Body Temperature

Body temperature, as an important physiological indicator, can largely reflect the user’s health status. Next, we introduce the flexible sensors used in wearable devices for monitoring body temperature. To enhance the response speed of temperature sensors, the industry has employed electrically sprayed inkjet printing technology to create temperature-sensitive inks [77]. This enables the device to react more swiftly to changes in the user’s body temperature, although there is a slight compromise in precision [47]. In practical applications, the sensor can be integrated into medical wearable devices to monitor patients’ body temperature. For athletes undergoing high-intensity training, an elevated body temperature may indicate excessive training load or muscle damage [78]. Therefore, it is also essential to use wearable devices containing this sensor for athletes.

### 4.4. Sensors Capable of Analyzing the Components of Body Fluids

**Figure 4:**
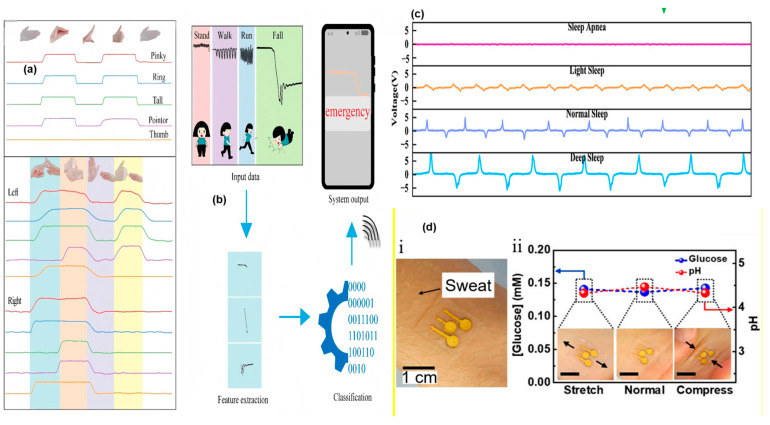
The diagram for some biosignals detection. (**a**) The different responses of sensor on different finger movements [6]. (**b**) Smart system for fall warning [6]. (**c**) Sweat analysis with mechanical deformation [79]. (**d**) Different curves within different sleep states [25].

Many uncommon physiological indicators also have significant reference value in the health field, such as body fluids [79]. Flexible sensors based on electrochemical principles can identify many types of body fluids, such as tears, saliva, and sweat. Amperometric sensors can determine the concentration of specific organic compounds by monitoring the current/voltage output curves. However, these output curves may contain noise originating from other organic substances in the sample. Through extensive control experiments, the noise can be eliminated from the overall curve. Currently, the vast majority of biosensors based on electrical principles are capable of detecting pH values and various ions (F^−^, I^−^, CN^−^, Na^+^, K^+^, etc.) [79]. The above details can be seen in Figure 4.

## 5. The Application of New Materials in Flexible Sensors

The development of new materials is the physical basis for innovative breakthroughs in flexible sensors. Over the past few years, nanomaterials and nanostructures, graphene-based materials, metals, and inorganic materials have all provided significant support for the innovation of flexible sensors [3,20,74,76,80,81,82]. In this section, we introduce the contributions of these materials to the development of flexible sensors.

### 5.1. Applications of Nanomaterials in Flexible Sensors

In flexible sensors, the fabrication of electrodes typically employs conventional conductive materials, such as liquid metals and silver nanowires. However, these materials are not optimal in terms of stretchability and rigidity. Next, we introduce a new type of flexible sensor. This sensor is composed of a super-elastic polyacrylamide (PAAm)/polyvinylpyrrolidone (PVP)/calcium chloride (CaCl_2_) conductive hydrogel and a surface-modified silicone rubber film. By increasing surface topography (e.g., surface roughness and surface area) and constructing various surface microstructures, its sensitivity, stretchability, and durability as a tactile sensor are significantly enhanced [83].

Meanwhile, due to the high dielectric constant of electrospun polyvinylidene fluoride (PVDF) nanofibers and the large contact area of the triboelectric layer, combined with a triboelectric layer featuring a hybrid micro-gold-tower structure with wrinkles, the sensor’s optimal output voltage is approximately 4.3 times that of the unmodified device [21].

### 5.2. Applications of Graphene Materials in Flexible Sensors

Next, we introduce the application of graphene materials in flexible sensors. The single-atom thickness and hexagonal honeycomb structure of graphene endow it with an extremely high surface area-to-volume ratio, as well as excellent electrical conductivity and mechanical properties [84,85], making it highly suitable for ultrasensitive detection applications [31].

Graphene quantum dots (GQDs) exhibit excellent biocompatibility, catalytic capability, and the ability to simultaneously detect multiple biomolecules. GQDs share similar morphological characteristics with graphene but have one or more panels on their sides, appearing spherical, which can provide numerous binding sites for biomolecules [31].

However, the mass production of graphene still faces challenges, and the use of graphene oxide (GO) is one of the most feasible solutions to address this issue [74]. Flexible pressure sensors made from commercial silk fabric and yarn coated with reduced graphene oxide (rGO), known as rGOS, exhibit high sensitivity (2.58 × 10^3^ kPa^−1^ at low pressure and 1.842 kPa^−1^ at high pressure). The GO coating is applied using a simple dip-coating method, followed by thermal reduction in the GO-coated silk (GOS) at 400 °C. The sensors can be fabricated by stacking small pieces of rGOS fabric and twisting rGOS yarns [74].

### 5.3. Applications of Inorganic and Metallic Materials

Inorganic and metallic materials have also played significant roles in the field of flexible sensors. For example, materials such as polyimide, polyurethane, and metallic silver [86,87].

**Figure 5:**
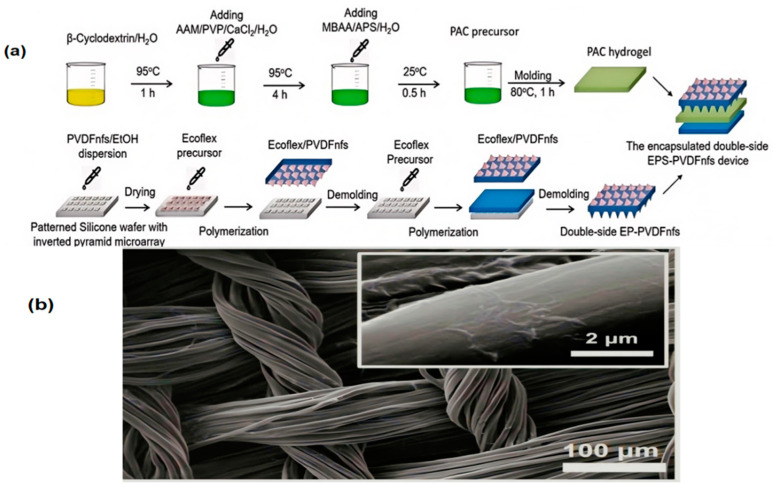
The diagram for some mechanism of materials: (**a**) steps in the fabrication of the double-side EPS-PVDFnfs device [21]; (**b**) shows GO samples coated onto the surface of the silk [74].

Conventional metallic materials may experience reduced conductivity due to cracking under severe strain. To address this issue, incorporating silver nanowires (AgNWs) into commonly used metallic thin films as elastic conductive bridges helps maintain the high conductivity of strain sensors under stretching and bending loads [30,32,88]. Ag particles are observed around the exposed AgNWs, which increases the contact area to enhance conductivity and reduce the NW-NW contact resistance. Moreover, the structure remains stable after 1000 bending cycles [32]. How to further enhance the adhesion of AgNWs to the substrate is a matter of significant interest. Next, we introduce a flexible pressure sensor made of two face-to-face AgNW-PI layers, where the AgNW-PI layer serves as the pressure-sensing element. By mixing Ag nanowires with PI polymer to form an AgNW-PI composite, the issue of poor adhesion is avoided. This pressure sensor features high sensitivity, flexibility, and low cost (PI stands for polyimide) [30]. Some of the details can be seen in Figure 5.

## 6. Conclusions and Challenges

After reviewing the latest progress in various aspects of flexible sensors in this paper, it is evident that the primary application fields of flexible sensors are in the detection of biological signals and health protection. Currently, the iteration speed of wearable devices in the market is accelerating. The durability, innovativeness, and performance of these products are all improving rapidly. However, the main challenges faced are the further improvement of biocompatibility, harmlessness, and miniaturization. Enhancements in these areas are inseparable from new advancements in microelectronics and electronic materials. The innovation of wearable devices is a systematic science and an emerging interdisciplinary field, integrating the latest achievements from medicine, microelectronics, communication engineering, materials science, and other disciplines. This paper looks forward to the updated achievements and further development of flexible sensors in the field of wearable devices.

## Data Availability

The original contributions presented in the study are included in the article, further inquiries can be directed to the corresponding author.
